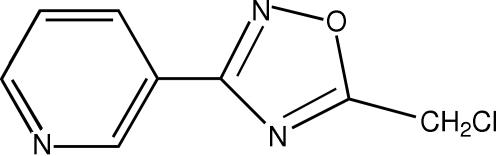# 4-[3-(Chloro­meth­yl)-1,2,4-oxadiazol-5-yl]pyridine. Corrigendum

**DOI:** 10.1107/S1600536808007058

**Published:** 2008-04-16

**Authors:** Si-shun Kang, Hai-Lin Li, Hai-su Zeng, Hai-bo Wang, Pin-liang Wang

**Affiliations:** a College of Science, Nanjing University of Technology, Xinmofan Road No.5 Nanjing, Nanjing 210009, People’s Republic of China

## Abstract

The title and the chemical diagram of the paper by Kang, Li, Zeng, Wang & Wang [*Acta Cryst.* (2007), E**63**, o4654] are corrected.

## Abstract 2

Corrigendum to *Acta Cryst.* (2007), E**63**, o4654.

In the paper by Kang, Li, Zeng, Wang & Wang [*Acta Cryst.* (2007), E**63**, o4654], the title and the chemical diagram are incorrect. The correct structure is shown below and the correct title of the original paper should be ‘3-[5-(Chloro­meth­yl)-1,2,4-oxa­di­az­ol-3-yl]pyridine’.